# CORONA-Net: Diagnosing COVID-19 from X-ray Images Using Re-Initialization and Classification Networks

**DOI:** 10.3390/jimaging7050081

**Published:** 2021-04-28

**Authors:** Sherif Elbishlawi, Mohamed H. Abdelpakey, Mohamed S. Shehata, Mostafa M. Mohamed

**Affiliations:** 1 Department of Computer Science, Math, Physics, and Statistics, The University of British Columbia, 3333 University Way, Kelowna, BC V1V 1V7, Canada; mohamed.abdelpakey@ubc.ca (M.H.A.); mohamed.sami.shehata@ubc.ca (M.S.S.); 2 Electrical and Computer Engineering Department, University of Calgary, Calgary, AB T2N 1N4, Canada; mabdelal@ucalgary.ca; 3 Biomedical Engineering Department, Helwan University, Cairo 11795, Egypt

**Keywords:** CORONA-Net, deep learning, COVID-19, pandemic

## Abstract

The COVID-19 pandemic has been deemed a global health pandemic. The early detection of COVID-19 is key to combating its outbreak and could help bring this pandemic to an end. One of the biggest challenges in combating COVID-19 is accurate testing for the disease. Utilizing the power of Convolutional Neural Networks (CNNs) to detect COVID-19 from chest X-ray images can help radiologists compare and validate their results with an automated system. In this paper, we propose a carefully designed network, dubbed CORONA-Net, that can accurately detect COVID-19 from chest X-ray images. CORONA-Net is divided into two phases: (1) The reinitialization phase and (2) the classification phase. In the reinitialization phase, the network consists of encoder and decoder networks. The objective of this phase is to train and initialize the encoder and decoder networks by a distribution that comes out of medical images. In the classification phase, the decoder network is removed from CORONA-Net, and the encoder network acts as a backbone network to fine-tune the classification phase based on the learned weights from the reinitialization phase. Extensive experiments were performed on a publicly available dataset, COVIDx, and the results show that CORONA-Net significantly outperforms the current state-of-the-art networks with an overall accuracy of 95.84%.

## 1. Introduction

The COVID-19 pandemic has affected most countries in 2019–2021. At the time of writing, there have been more than 122 million confirmed cases of COVID-19 and over 2 million confirmed deaths resulting from the COVID-19 disease worldwide. As such, governments around the world are trying to minimize the spread of the virus until a reliable cure or vaccine can be discovered. As the virus has a very high transmission rate, people have been practicing social distancing to reduce the chance of catching the disease. Doctors also need a way to rapidly test patients for COVID-19, so that they can begin treating them if they have the disease. There are two types of tests for COVID-19, namely viral and antibody tests. The viral test checks samples from the respiratory system for SARS-CoV-2, the virus that causes COVID-19 disease. This type of test can be performed in an hour or two, but in some cases it can take up to 1–2 days to obtain results if the test has to be sent to a laboratory. The viral test only indicates if a current infection exists, but not if there was an old infection. On the other hand, the antibody test is performed by taking a blood sample and can detect if there was a previous infection with the SARS-CoV-2 virus, even if there are no current symptoms.

**Figure 1:**
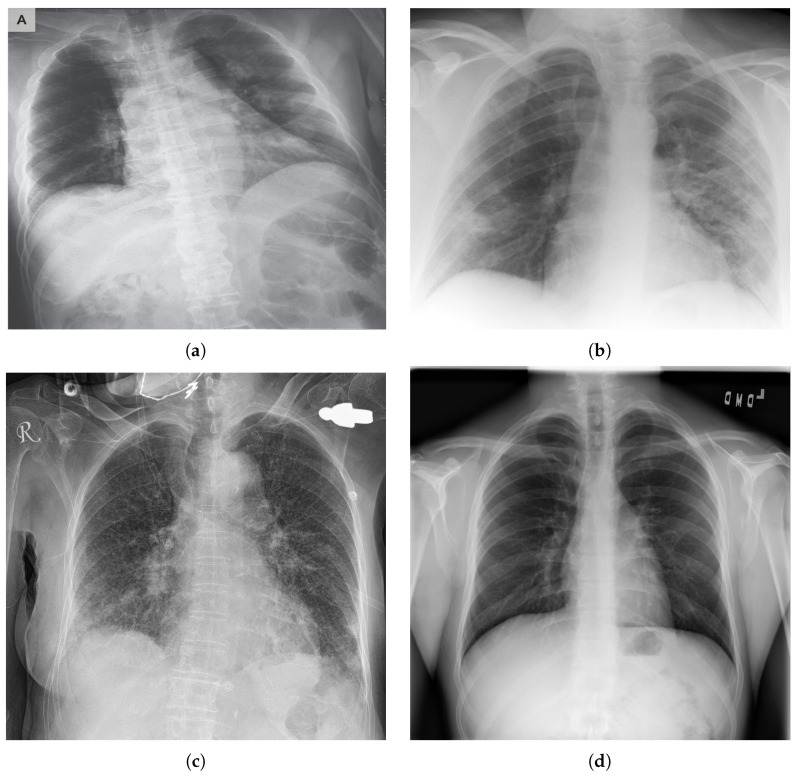
Chest X-ray of a person (**a**) with a COVID-19, (**b**) with COVID-19 pneumonia, (**c**) with pneumonia, and (**d**) a normal case.

COVID-19 typically causes pneumonia in human lungs, which can be detected in X-ray images (see Figure 1). Datasets of people with pneumonia inflicted by COVID-19, as well as people with pneumonia inflicted by other diseases, became available online, thus enabling the possibility to create deep learning networks that can differentiate between X-ray images of people with COVID-19 and people who do not have the disease. The dataset used in this study is publicly available and can be accessed online (https://github.com/ieee8023/covid-chestxray-dataset (accessed on 12 February 2021)). Using deep learning can help save time in testing for COVID-19 in time-critical situations, which could potentially save more lives. Having results obtained from AI can help clinicians validate their results as well as make better-informed decisions. The structure of this paper is as follows:

- Firstly, we propose CORONA-Net, a novel deep learning architecture. CORONA-Net has two phases: (1) The reinitialization phase and (2) the classification phase. In the reinitialization phase, the network learns a prior distribution that is suitable for medical images. In the classification phase, the network classifies the input image to one of three classes: COVID-19, Pneumonia, and Normal.
- Secondly, the proposed network is trained using a decoder network in the reinitialization phase, then the decoder network is discarded. This procedure of learning allows CORONA-Net to learn a prior distribution that encodes the medical images’ distribution instead of natural images.
- Finally, the design of the proposed network increases the sensitivity to the COVID-19 class.

The novelty of this paper can be summarized as follows: (1) The network is designed to leverage the limited available data for COVID19 by introducing the reinitialization phase. The reinitialization phase is used to reinitialize the weights of the network by a distribution of medical images instead of natural images. (2) The classification phase is built upon the reinitialization phase so that the network can be optimized using the cross-entropy loss function for medical images’ distribution without using natural images’ distribution. The source code and results of the proposed network in this paper have been made publicly available (https://vip-mun.github.io/CORONA_NET/ (accessed on 26 April 2021)).

The remainder of the paper is organized as follows. We first introduce the related work in Section 2. Section 3 details the proposed CORONA-Net formulation and training along with the classification phase. The ablation study and experimental results are described in Section 4. Finally, Section 5 concludes the paper.

## 2. Related Works

Using deep learning [1] to test for patients with COVID-19 was proposed in recently published work that uses CT images to detect COVID-19 [2,3,4,5]. However, these research efforts are closed-source and are not open to the research community for contribution and comparison. Fortunately, the research community has been trying to publish datasets of COVID-19 in the public domain so that more research can be done more easily and rapidly in this field. Thus, the COVID-19 Image Collection dataset was created with the help of online contributors at GitHub for data collection [6]. This has led to many research contributions in the field of detecting COVID-19 from X-ray and CT images [4,7,8,9,10,11,12,13,14]. Some of these publications have promising results and the accuracy increases as the COVID-19 dataset grows.

Wang et al. [15] proposed the Inception network to extract features from patients to compute tomography for image scans. Using a plain inception network, this method was able to produce some promising results with an accuracy over 80%. Al-antari et al. [16] predicted COVID-19 cases from digital chest X-ray images using a computer-aided diagnose network based on the YOLO predictor [17]. The network consists of a CNN-based deep feature extraction followed by fully connected layers that then gives a confidence score that can tell if the X-ray belongs to the COVID-19 class. Asif et al. [18] used a Deep Convolutional Neural Network (DCNN) to achieve the same task. DCNN is based on Inception-V3 with transfer learning and this network achieved over 90% accuracy for detecting COVID-19 from X-ray images. Arora et al. [19] utilized DenseNet and GoogleNet to detect COVID-19 from X-ray images. The network takes the input image then takes a multi-path approach, where one path goes through the DenseNet block, while the other goes through the GoogleNet block. The output of both paths is combined and followed by a variational auto-encoder and a classifier. The final output is one of the three classes COVID-19, Pneumonia, or Normal.

Hemdan et al. [20] proposed a deep network, namely COVIDX-Net, to classify COVID-19 images from non-COVID-19 images. They used a dataset of 50 chest X-ray images. Seven networks were used for feature extraction and all the results were compared to determine the optimal feature extractor network to achieve this task. The highest performing feature extractors were VGG-19 and DenseNet201, which achieved over 90% accuracy in classifying COVID-19 images. Narin et al. [21] compared the results of three deep networks to detect COVID-19 in chest X-ray images, namely ResNet-50, Inception-V3, and Inception-V2. After training on the same dataset with all three networks, ResNet-50 achieved the best performance with an accuracy of over 98%. Pereira et al. [22] proposed a classification network based on the classic Convolutional Neural Network (CNN) and popular texture descriptors. A resampling algorithm was used to balance the training dataset for the multiple classes of the classification problem and achieved an F1-score of 65%. The work proposed by Apostolopoulos et al. [23] used transfer learning to detect abnormalities in medical images. The authors used different network architectures to select the best performer. Furthermore, the method collected medical images for COVID-19 from publicly available repositories. A Similar work proposed by Brunese [24] utilized transfer learning using the VGG-16 network to build two networks. The first network was responsible for classifying the image based on whether the person is healthy or not. In case the image was classified as unhealthy, the second network took the image and classified whether the person has COVID-19 or not. Hu et al. [25] proposed a weakly supervised deep framework to detect COVID-19 from CT images. The framework used VGG-16 for feature extraction and multi-scale learning to tackle the problem of lesion scattering and size variations.

Ouchicha et al. [26] proposed COVIDNet, a CNN-based model to classify COVID-19 infections from normal and other pneumonia cases using chest X-ray images. It is built by using two parallel levels with differently sized kernels to detect local and global features of the inputs. CVDNet was trained on a small dataset that contained only 219 COVID-19 chest X-ray images but produced promising results.

Yoo et al. [27] investigated a deep-learning-based decision-tree classifier for detecting COVID-19 from chest X-ray images. The classifier consists of three binary decision trees, which are trained on a CNN-based network made with PyTorch. The first decision tree classifies the image as normal or abnormal. The second tree looks at the abnormal images and checks if they have any sings of tuberculosis. Finally, the third decision tree checks the abnormal images for COVID-19. The paper achieved a good COVID-19 accuracy detection of 95%.

Caobelli et al. [28] proposed COVIDX-Net, a model consisting of seven CNN models for COVID-19 detection from chest X-ray images. Sethy et al. [29] classified CNN features with Support Vector Machine (SVM) using chest X-ray images. The paper concluded that the ResNet50 model with the SVM classifier provided the best performance in comparison to other networks tested. Other recent papers used various deep learning methods with CT images, such as [30,31,32,33].

Most of these methods use transfer learning from a network that has been trained on natural images. We argue that the underlying pattern and distribution of medical images are different from natural images. Therefore, the proposed method has a reinitialization phase to ensure that the backbone network captures the underlying patterns and distribution of medical images.

## 3. Materials and Methods

**Figure 2:**
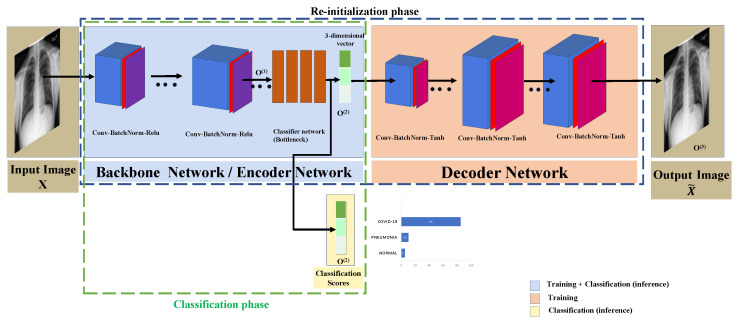
CORONA-Net architecture: the backbone network and the fully connected layers are shown in the blue block, the decoder network is shown in the orange block, and the confidence score is highlighted in yellow.

CORONA-Net consists of two phases, namely the reinitialization phase and the classification phase. The CORONA-Net architecture is motivated by the fact that there are no networks pre-trained on large medical datasets such as ImageNet [34] to use their weights to transfer the knowledge from one task to another. To initialize the network from medical image distribution, the reinitialization optimization phase is proposed. Once the network has been initialized, the classification phase starts the training process from the weights that were learned from the reinitialization phase. The backbone network of CORONA-Net is VGG16 [35], which acts as an encoder in the reinitialization phase and a feature extractor in the classification phase. The backbone network has four linear layers that output a three-dimensional vector that acts as a bottleneck layer of the encoder in the reinitialization phase. Similarly, the three-dimensional vector phase acts as the confidence score of the classifier in the classification phase. In the reinitialization phase, the four linear layers are followed by the decoder network to upsample the three-dimensional vector to the same input image size, as shown in Figure 2. In the classification phase, CORONA-Net gets rid of the decoder network and the whole network acts as a classifier.

### 3.1. Re-Initialization Optimization Phase

In this phase, the backbone network and the four linear layers are used as an encoder to encode the input image *X* to a compressed three-dimensional feature vector. Furthermore, the decoder network is used to output an image similar to the input image. In this phase, the training is done by feeding a batch of images (i.e., 50 images) to the CORONA-Net; the input image has a spatial size of 224×224×3. The image is passed through two convolutional layers. The next layers consist of two convolutional layers of the same size. These layers are followed by a max-pooling layer to output a down-sampled feature map, then two convolutional layers of the same size and a max-pooling layer, followed by another convolutional layer with the output of features maps of depth = 256. The whole architecture’s data dimension is shown in Table 1 and Table 2. The final output of the encoder network is a three-dimensional vector that is a compressed version of the input image with a high-level representation. On the other hand, the final output of the decoder network is a tensor of size (N,3,224,224) because the decoder network outputs the same spatial size as the input tensor to the encoder network, which is in this case the input image. Note that N = 50 is the batch size.

The three-dimensional feature vector is then fed into the decoder network to generate an image $\bar{X}$. For example, the input image to the CORONA-Net is *X* with a spatial size of 224×224×3, and the output of the decoder is 224×224×3 as well. The decoder network architecture consists of 12 convolutional layers, where each layer is followed by a batch normalization layer and a tanh non-linearity. The tanh non-linearity, in this case, works better than ReLU because the output of the decoder network is an RGB image and not a probability. Moreover, ReLU always thresholds the input values to less than zero. It is worth mentioning that CORONA-Net does not care about the quality of the output image of the decoder network, as the ultimate goal of the decoder network is to help initialize the backbone network with weights that have been optimized for medical images’ distribution. It is worth mentioning that the spatial size of each layer in the decoder network is selected to ultimately produce an output of the same input’s spatial size, as shown in Table 2. Consequently, the design of the reinitialization network aims at trying to learn an approximation to the identity function. This approximation can be learned by optimizing the networks to construct an output image that is similar to the input image. After training the encoder and decoder networks, the decoder network is removed from CORONA-Net. Consequently, the initialized backbone (i.e., the encoder) network is no longer an identity function. This phase is important so as to initialize CORONA-Net by using the medical images’ distribution instead of natural images. This design forces CORONA-Net to learn a compressed representation of the input image represented by the output of the four linear layers (bottleneck). The reinitialization phase is required because the available data of COVID-19 is quite limited at the time of writing this paper. The spatial size of each block in the decoder network is shown in Table 2.

### 3.2. Classification Phase

After the reinitialization phase, the decoder network is separated out from CORONA-Net. Now the backbone/encoder network is initialized with weights that come from medical images’ distribution rather than natural images.

In this setting, the input image in the batch is fed into the backbone network (i.e., the encoder network) to go through the two convolutions layers with batch normalization and ReLU followed by a max-pooling layer. Then, the output of the previous layers is fed into two convolution layers with batch normalization and ReLU followed by three convolution layers and a max-pooling layer, etc. All layers are listed in Table 1. It is worth noting that the backbone network (i.e., the encoder network) uses the ReLU non-linearity instead of the tanh non-linearity. The reason behind choosing the ReLU over tanh is the nature of the classification, where the output is a probability and it should always be positive. This is unlike the decoder network, where the output is an image and it needs to maintain positive and negative information. This includes convolutions, batch normalization, and ReLU layers as shown in Table 1. Then, the classifier network shown in Figure 2 will output the classification score $O^{\left(2\right)}$ for each class in {COVID-19, Pneumonia, Normal}. The classification score $O^{\left(2\right)}$ is a three-dimensional vector. Note that the three-dimensional vector in the reinitialization phase $O^{\left(2\right)}$ is the same three-dimensional vector used in the classification phase, as shown in Figure 2. An Adam optimizer is used to maximize the likelihood between the predicted classification score and the ground-truth labels of the corrected classes. It is worth mentioning that the COVID-19 class encodes all people who have SARS-CoV-2, the Pneumonia class encodes all people who have pneumonia due to any virus, and the Normal class encodes the normal people who do not show any symptoms.

### 3.3. CORONA-Net

The backbone network and the decoder network act as an auto-encoder in the reinitialization phase to output an RGB image $\bar{X}\in R^{224\times 224\times 3}$ that is similar to the input image $X\in R^{224\times 224\times 3}$.

#### 3.3.1. In the Reinitialization Phase

CORONA-Net is trained in an unsupervised learning manner (i.e., no actual labels) that works by applying the back-propagation algorithm to set the output spatial size of the network to be similar to the input spatial size. Therefore, CORONA-Net essentially is trying to learn an approximation to the identity function at the reinitialization phase. This function always returns an output image that looks very similar to the input image. Both the backbone network and the decoder network formulate the learned identity function that has been optimized for the medical images. Once the reinitialization phase has been trained, CORONA-Net removes the decoder network. Consequently, the backbone network has powerful initialization weights that are optimized for medical images.

Let ϕ denote the backbone network, and the output of the backbone network is given by the following equation:

(1) $$\phi \left(X\right)=O^{\left(1\right)}$$

where $X\in R^{224\times 224\times 3}$ is the input image and O(.) is the backbone network output. The output of the backbone network (i.e., the four linear layers) is a lower representation of a three-dimensional vector. Let β denote the fully connected layers and the output of this network be given by:

(2) $$\left(O^{\left(1\right)}\right)=O^{\left(2\right)}$$

where $O^{\left(2\right)}\in R^{3\times 1}$ is a three-dimensional feature vector output of the fully connected layers. Consequently, $O^{\left(2\right)}$ is then fed into the decoder network. Let γ denote the decoder network and the output of the decoder network be:

(3) $$\left(O^{\left(2\right)}\right)=O^{\left(3\right)}$$

where $O^{\left(3\right)}\in R^{224\times 224\times 3}$ is the final output of CORONA-Net in the reinitialization phase. In this phase, CORONA-Net minimizes the loss function between the output of the decoder and the input image in an unsupervised fashion using the mean squared error as follows:

(4) $$L\left(O^{\left(3\right)},X\right)=\frac{1}{N}\sum_{i=1}^{N}{\left(O^{\left(3\right)}-X\right)}^{2}$$

where *N* is the batch size. It is worth noting that the aforementioned equations (i.e., before the loss function) assume that there is a batch size of 1; however, the assumption is true for N>1.

#### 3.3.2. In the Classification Phase

As mentioned before, in the classification phase, the decoder network is removed from CORONA-Net. In this phase, the output of fully connected layers is a three-dimensional classification score vector that encodes the confidence of each class (i.e., COVID-19, Pneumonia, and Normal). The training in this phase is done using the actual labels of the COVIDx dataset [36] in a fully supervised manner. Therefore, the network can classify with a high confidence score if a chest X-ray/CT image belongs to the COVID-19, Pneumonia, or Normal (a healthy person) class. In this phase, CORONA-Net is trained using the cross-entropy loss as follows:

(5) $$L\left(O^{\left(2\right)},y\right)=-\frac{1}{N}\sum_{i=1}^{N}\left(y_{i}log\left(S\left(O^{\left(2\right)}\right)\right)\right)$$

where *y* is the ground truth labels in the batch and $y_{i}$ is the label of image *i* in the batch formed in a one-hot-vector, a vector with all zeros except one element labeled by “1” to indicate the location of the correct class, *S* is the Softmax that normalizes the output of the network, and $O^{\left(2\right)}$ is the output of the fully connected layers. It is worth mentioning that both phases are optimized by an Adam optimizer with a starting learning rate of $10^{-4}$ in the reinitialization phase and $3\times 10^{-4}$ in the classification phase.

## 4. Experimental Results

**Dataset:** CORONA-Net was trained on the COVID-19 image collection dataset [6,36], namely COVIDx [36]. The dataset is publicly available and includes chest X-rays images and CT images. Furthermore, the COVIDx dataset has the biggest collection of COVID-19 X-rays and CT images (at the time of writing) as well as SARS, MERS, and ARDS. Data are collected from public sources and are updated on a regular basis as more images become available from medical labs. The COVID-19 image collection dataset has five sub-classes, including the Pneumonia, COVID-19, SARS, *Streptococcus* spp., *Pneumocystis* spp. and ARDS classes. The dataset was curated to reduce the number of classes to three, namely COVID-19, Pneumonia, and Normal, and renamed COVIDx. In general, COVIDx has a total number of 13,962 images for 13,870 patient cases. Specifically, COVIDx contains 358 images that were taken from 266 positive cases of COVID-19. Moreover, the Normal class contains 8066 images that were taken from normal people who did not have any disease and 5538 images for cases with pneumonia.

### 4.1. Results

**Figure 3:**
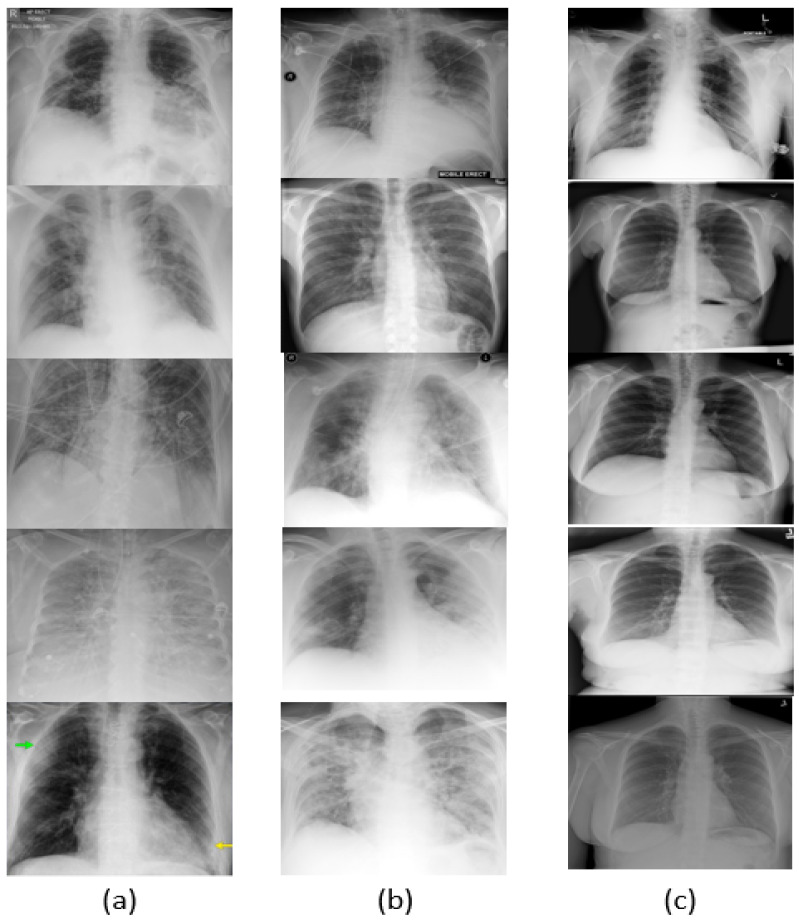
Visual results. (**a**) Images classified as COVID-19, (**b**) Images classified as pneumonia, and (**c**) Images classified as normal.

**Figure 4:**
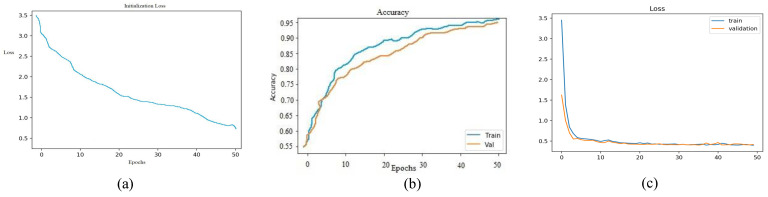
Performance of CORONA-Net (**a**) Error of the loss function on the initialization phase. (**b**) Accuracy of CORONA-Net after training. (**c**) Error of the loss function on the classification phase.

Since COVID-19 is a recent disease, the dataset is still quite limited, and thus data augmentation is needed to obtain more varied samples. For data augmentation, image transformations such as image resizing, random horizontal flipping, random rotation, and color jittering were applied to the input batches. The visual results are shown in Figure 3. As shown in Figure 4a–c, the network was trained over 50 epochs, after which it reached the the maximum accuracy (i.e., overall accuracy) of 95.084%. Figure 4a,b also show that 50 epochs are sufficient for training. Note that the loss curve plateaus after 50 epochs, and therefore we plot the curves for 50 epochs. The loss curves of the training and validation of the reinitialization phase and classification phase are shown in Figure 4c.

**Figure 5:**
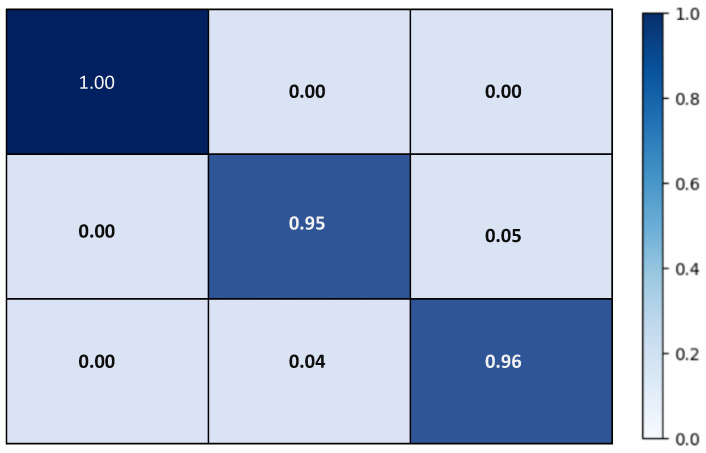
Confusion Matrix of CORONA-Net on COVIDx test dataset.

After training was complete, the results on the testing set were obtained and can be seen in Figure 5. CORONA-Net achieved 100% sensitivity to the COVID-19 class, 95% sensitivity in classification of the Pneumonia class and 95% sensitivity in classification of the Normal class. These results show that CORONA-Net gives a highly accurate prediction with the most sensitivity to the COVID-19 class. This is important, as detecting/classifying this class accurately is more important than other classes.

A summary comparison of CORONA-Net against state-of-the-art methods is shown in Table 3. Table 3 quantifies each network in terms of Positive Predictive Value (PPV). PPV is defined as the proportion of predicted positives that are actual positives. CORONA-Net outperformed all other networks in terms of PPV on the three classes COVID-19, Pneumonia, and Normal. CORONA-Net had a gain of 9.5% compared to the second best, which had a PPV of 90.5% on the COVID-19 class. By looking at the Pneumonia class, one can clearly see that there was a gain of 5.2% in favor of CORONA-Net. It is worth mentioning that CORONA-Net was more accurate for both the COVID-19 and Pneumonia classes than for the Normal class. Table 4 shows the $F_{1}$ score for each class; it can be observed that the COVID-19 class had the highest $F_{1}$ score of 99.8. Moreover, Table 5 shows the comparison of CORONA-Net against state-of-the-art methods in terms of accuracy.

### 4.2. Ablation Study

To show the impact of the different components in the CORONA-Net architecture, more experiments were conducted. An experiment was conducted to show the role of the reinitialization phase along with the pre-trained backbone. The pre-trained backbone in Table 6 shows the effect of using the pre-trained backbone network with ImageNet. By using the pre-trained weights the sensitivity for each class increased; for example, the COVID-19 class had a gain of 19% while the Normal class increased from 55% to 62%. Consequently, adding pre-trained weights of ImageNet helped the network gain moderate performance.

On the other hand, if CORONA-Net was trained with a reinitialization phase and without the pretrained weight, the sensitivity increased significantly; for example, the COVID-19 class increased from 44% to 93% with a gain of 49%. The reinitialization phase increased the sensitivity of the Pneumonia class as well by a gain of 38%. This shows the importance of adding the reinitialization phase to CORONA-Net, and it also confirms that it is important to start training with a pre-initialized network with medical images, not natural images. There is a significant impact when adding the reinitialization phase in addition to the pre-trained weights, as the sensitivity for all classes increased dramatically to reach 100% in terms of sensitivity in the COVID-19 class, 95% in the Pneumonia class, and 95% in the Normal class. Here, we emphasize that the sensitivity to the COVID-19 class was 100%—only for this class and not the sensitivity over all classes, since the available number of images is quite limited compared to other classes (i.e., the Normal class and the Pneumonia class). Moreover, we also emphasize that data augmentation and regularization were employed in the training to avoid overfitting due to the imbalanced distribution of the number of images per class. As shown in Table 6, CORONA-Net had the most sensitivity to the COVID-19 class, which is a promising network design because it is more important to recognize people with COVID-19 than recognizing healthy people.

**Figure 6:**
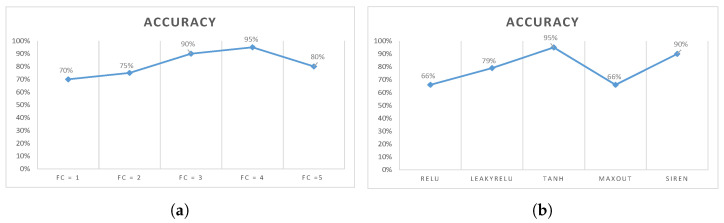
Impact of (**a**) adding fully connected layers to the classifier, (**b**) adding different activation functions.

Another experiment was conducted on the number of fully connected layers. The experiment started by adding layers one by one, up to five fully connected layers. Figure 6a shows that after adding one fully connected layer, the overall accuracy was 70% on the testing set. After adding one more layer, the accuracy increased by 5%. In this experiment, five layers were tested as mentioned. As shown in Figure 6a, the maximum accuracy was obtained at the number of fully connected layers (FC) = 4, and the accuracy dropped down by 15% when adding the fifth layer. Based on this experiment, four layers were selected in the design of CORONA-Net.

One of the key design considerations for CORONA-Net is the activation function in the decoder network. To show the effect of using different activation functions on CORONA-Net, another experiment was conducted. Figure 6b shows that five different activation functions, namely ReLU [34], LeakyReLU [37], tanh, MaxOut [38], and the recently proposed SIREN [39] were tested on CORONA-Net. It is clear that different activation functions have different impacts on CORONA-Net. For example, even though ReLU worked better in most scenarios, tanh outperformed ReLU with a gain of 29% and SIREN outperformed it with a gain of 5% in this design. One of the most important reasons to use the tanh in the decoder network in this design is because it maintains the positive and negative information and has a wider range of activation [−1,+1]. Moreover, the output of tanh is more dense, which is essential information needed to construct the output image from a low-dimensional input.

## 5. Conclusions

In this paper, a novel network design was proposed. Unlike most network designs in the literature, the proposed CORONA-Net has two phases: (1) A reinitialization phase and (2) a classification phase. The reinitialization phase was designed to force the weights of the backbone/encoder network to be more suitable for medical images. The proposed CORONA-Net architecture substantially increases the sensitivity and Positive Predictive Value (PPV) of predictions over the classes. Consequently, CORONA-Net can have a significant and positive impact on the health-care systems as testing every person suspected of having the disease is difficult. After training, CORONA-Net provided accurate and promising results in terms of sensitivity, PPV, and overall accuracy. We hope that our work can be used to more easily test for COVID-19 and contribute to helping bring this pandemic to an end.

## Figures and Tables

**Table 1:** Data dimensions in CORONA-Net (the backbone/encoder network).

Layers	Kernel Size	Stride	Padding	Output Channel
Input	Tensor (50, 3, 224, 224 )	-	-	-
Convolution (BatchNorm+Relu)	K = 3 × 3	S = 1	P = 1	O = 64
Convolution (BatchNorm+Relu)	K = 3 × 3	S = 1	P = 1	O = 64
MaxPooling	K = 2 × 2	S = 2	P = 0	-
Convolution (BatchNorm+Relu)	K = 3 × 3	S = 1	P = 1	O = 128
Convolution (BatchNorm+Relu)	K = 3 × 3	S = 1	P = 1	O = 128
MaxPooling	K = 2 × 2	S = 2	P = 0	-
Convolution (BatchNorm+Relu)	K = 3 × 3	S = 1	P = 1	O = 256
Convolution (BatchNorm+Relu)	K = 3 × 3	S = 1	P = 1	O = 256
Convolution (BatchNorm+Relu)	K = 3 × 3	S = 1	P = 1	O = 256
MaxPooling	K = 2 × 2	S = 2	P = 0	-
Convolution (BatchNorm+Relu)	K = 3 × 3	S = 1	P = 1	O = 512
Convolution (BatchNorm+Relu)	K = 3 × 3	S = 1	P = 1	O = 512
Convolution (BatchNorm+Relu)	K = 3 × 3	S = 1	P = 1	O = 512
MaxPooling	K = 2 × 2	S = 2	P = 0	-
Convolution (BatchNorm+Relu)	K = 3 × 3	S = 1	P = 1	O = 512
Convolution (BatchNorm+Relu)	K = 3 × 3	S = 1	P = 1	O = 512
Convolution (BatchNorm+Relu)	K = 3 × 3	S = 1	P = 1	O = 512
Convolution	K = 1 × 1	S = 1	P = 0	O = 100

**Table 2:** Data dimensions in CORONA-Net (the decoder network).

Layers	Kernel Size	Stride	Padding	Output Channel
ConvolutionTranspose (BatchNorm+Tanh)	K = 2 × 1	S = 1 × 1	P = 0	O = 6
ConvolutionTranspose (BatchNorm+Tanh)	K = 2 × 2	S = 2 × 1	P = 0	O = 8
ConvolutionTranspose (BatchNorm+Tanh)	K = 2 × 2	S = 2 × 2	P = 0	O = 10
ConvolutionTranspose (BatchNorm+Tanh)	K = 3 × 3	S = 1 × 1	P = 0	O = 14
ConvolutionTranspose (BatchNorm+Tanh)	K = 4 × 4	S =4 × 4	P = 0	O = 16
ConvolutionTranspose (BatchNorm+Tanh)	K = 3 × 3	S = 3 × 3	P = 0	O = 18
ConvolutionTranspose (BatchNorm+Tanh)	K = 3 × 3	S = 2 × 2	P = 0	O = 22
ConvolutionTranspose (BatchNorm+Tanh)	K = 3 × 3	S = 1 × 1	P = 4	O = 24
ConvolutionTranspose (BatchNorm+Tanh)	K = 3 × 3	S = 1 × 1	P = 4	O = 32
ConvolutionTranspose (BatchNorm+Tanh)	K = 3 × 3	S = 1 × 1	P = 4	O = 3
ConvolutionTranspose (BatchNorm+Tanh)	K = 4 × 4	S = 1 × 1	P = 3	O = 3
Output	Tensor (50, 3, 224, 224)	-	-	-

**Table 3:** Comparison of positive predicted value with state-of-the-art methods.

Positive Predictive Value (%)			
Architecture	COVID-19	Pneumonia	Normal
VGG-19	83.1	75.0	98.4
ResNet-50	88.2	86.8	98.8
COVID-Net [36]	90.5	91.3	**98.9**
CORONA-Net (ours)	**100**	**96.5**	93.0

**Table 4:** $F_{1}$ score for CORONA-Net.

$F_{1}$ Score (%)			
	COVID-19	Pneumonia	Normal
CORONA-Net (ours)	**99.8**	**95.7**	**93.9**

**Table 5:** Results of CORONA-Net compared to state-of-the-art methods.

Method	Classes	Cases per Class	Type of Network	Accuracy (%)
Ioannis et al. [23]	COVID-19 (+)	224	VGG-19	93.48
	Pneumonia	700		
	Healthy	504		
Narin et al. [21]	COVID-19 (+)	50	ResNet 50	95
	COVID-19 (−)	50		
Wang and Wong [36]	COVID-19 (+)	53	COVID-Net	92.4
	COVID-19 (−)	5526		
	Healthy	8066		
Hemdan et al. [20]	COVID-19 (+)	25	COVIDX-Net	90.0
	Normal	25		
Sethy and Behra [29]	COVID-19 (+)	25	ResNet50	95.38
	COVID-19 (−)	25		
CORONA Net (ours)	COVID-19 (+)	358	VGG-16	**95.84**
	Pneumonia	8066		
	Healthy	5538		

**Table 6:** Ablation study using variations of CORONA-Net.

Variations of CORONA-Net			Sensitivity for Each Class		
Backbone Network	Reinitialization	Pre-Trained	COVID-19	Pneumonia	Normal
✓			44%	51%	55%
✓		✓	63%	68%	62%
✓	✓		93%	89%	88%
✓	✓	✓	100%	95%	95%

## Data Availability

https://vip-mun.github.io/CORONA_NET/ (accessed on 27 April 2021).
